# In vitro and in vivo anti-malarial activity of novel harmine-analog heat shock protein 90 inhibitors: a possible partner for artemisinin

**DOI:** 10.1186/s12936-016-1625-7

**Published:** 2016-12-01

**Authors:** Abebe Genetu Bayih, Asongna Folefoc, Abu Naser Mohon, Scott Eagon, Marc Anderson, Dylan R. Pillai

**Affiliations:** 1 Department of Pathology and Laboratory Medicine, MIID and Medicine, University of Calgary, Calgary, AB Canada; 2 Department of Medical Parasitology, College of Medicine and Health Sciences, University of Gondar, Gondar, Ethiopia; 3 Department of Chemistry and Biochemistry, California Polytechnic State University, San Luis Obispo, CA USA; 4 Department of Chemistry and Biochemistry, San Francisco State University, San Francisco, CA USA

**Keywords:** Malaria, *Plasmodium falciparum*, Anti-malarial drugs, Heat-shock protein 90

## Abstract

**Background:**

The emergence of artemisinin-resistant *Plasmodium falciparum* strains poses a serious challenge to the control of malaria. This necessitates the development of new anti-malarial drugs. Previous studies have shown that the natural beta-carboline alkaloid harmine is a promising anti-malarial agent targeting the *P. falciparum* heat-shock protein 90 (PfHsp90). The aim of this study was to test the anti-malarial activity of harmine analogues.

**Methods:**

Forty-two harmine analogues were synthesized and the binding of these analogues to *P. falciparum* heat shock protein 90 was investigated. The in vitro anti-malarial activity of two of the analogues, 17A and 21A, was evaluated using a 72-h growth inhibition assay. The in vivo anti-malarial activity was tested in *Plasmodium berghei* infection of BALB/c mice. The potential of 21A for a combination treatment with artemisinin was evaluated using in vivo combination study with dihydro-artemisinin in BALB/c mice. Cytotoxicity of the harmine analogues was tested in vitro using HepG2 and HeLa cell lines.

**Results:**

17A and 21A bound to PfHsp90 with average IC_50_ values of 12.2 ± 2.3 and 23.1 ± 8.8 µM, respectively. They also inhibited the *P. falciparum W2* strain with average IC_50_ values of 4.2 ± 1.3 and 5.7 ± 1.7 µM, respectively. In vivo, three daily injections of *P. berghei*-infected BALB/c mice with 100 mg/kg of either 17A or 21A showed significant reduction in parasitaemia with a 51.5 and 56.1% reduction, respectively. Mice treated with 17A and 21A showed a median survival time of 11 and 14 days, respectively, while the vehicle control mice survived a median of only 8.5 days. A dose-ranging experiment with 21A showed that the compound has a dose-dependent anti-malarial effect. Furthermore, treatment of infected mice with a combination of 21A and dihydroartemisinin (DHA) showed a dramatic reduction in parasitaemia compared to treatment with DHA alone.

**Conclusion:**

A novel and non-toxic harmine analogue has been synthesized which binds to PfHsp90 protein, inhibits *P. falciparum* in vitro at micromolar concentration, reduces parasitaemia and prolongs survival of *P. berghei*-infected mice with an additive anti-malarial effect when combined with DHA.

## Background

Malaria is one of the leading causes of morbidity and mortality from infectious diseases. A recent report by World Health Organization (WHO) estimated that 3.2 billion people are at risk of contracting the disease. In 2015 alone, there were an estimated 214 million cases of malaria worldwide with 438,000 deaths. Ninety per cent of the deaths occurred in WHO African Region where children are the main victims [1].

Multidimensional intervention in malaria control over the last decade has significantly reduced the incidence and mortality caused by malaria worldwide. These include the provision of insecticide-treated bed nets, indoor residual spraying, rapid diagnostic tests, and a supply of effective artemisinin-based combination therapy (ACT). As a result, many countries have initiated malaria elimination programmes. However, the emergence of *Plasmodium falciparum* strains that are resistant to artemisinin in Southeast Asian countries is posing a huge challenge to future malaria control and elimination efforts. The possibility of dissemination of the resistant strains to Africa is projected to have potentially catastrophic outcomes [1]. This calls for the urgent search for new and effective anti-malarial drugs.

Currently, ACT is the most widely used treatment for uncomplicated malaria. Since artemisinin is a fast-acting drug with a short half-life, it should be combined with another drug with longer half-life in order to effectively clear the parasite and prevent the emergence of drug-resistant strains. Several partner drugs have been used as a component of ACT. Unfortunately, treatment failures in the partner drugs have emerged, threatening to curb the positive achievements gained to date in the fight against malaria and exposing artemisinin [2–4]. This necessitates the development of new partner drugs to use in ACT in places where resistance to artemisinin has not developed.

Heat shock proteins (Hsps) are the major chaperone proteins found in all life forms, ranging from prokaryotes to higher organisms, such as plants and mammals. Hsps are both constitutive and stress-inducible [5, 6]. Environmental factors, such as an abrupt change in temperature, upregulates the expression of Hsps [7, 8].

The life cycle of parasites such as *Plasmodium*, *Leishmania* and *Trypanosoma* involves poikilothermic insect vectors and homoeothermic mammalian hosts. These parasites are exposed to a sudden change in temperature of up to 10 °C during the transition from the insect-stage to mammalian-stage of the parasites, and have evolutionarily developed molecular chaperones to withstand the drastic change in temperature [9, 10]: for example, about 2% of the genes of *P. falciparum* code for proteins that serve as molecular chaperones [9]. Su and Wellems [11] showed that transcription of *P. falciparum* heat shock protein 90 (PfHsp90) increases up to three- and four-fold as a result of in vitro cultivation of the parasite at 39 and 41 °C, respectively.

Depending on their molecular size, Hsps are classified as small heat shock proteins (sHsps), Hsp40, Hsp60, Hsp70, Hsp90, and Hsp110. Hsp90 is one of the most abundant cytosolic proteins of a eukaryotic cell. The N-terminal domain of Hsp90 has an ATP binding pocket responsible for its ATPase activity [12, 13]. Hsps play a crucial role in the normal metabolic activities of cells. By facilitating the proper folding of proteins, Hsps are involved in intracellular protein trafficking, gene expression, cell cycle, as well as cell differentiation [5, 9, 14].

The crucial role of Hsp90 in chaperoning several important cellular functions and the structural differences in the ATP-binding domain of human and parasite Hsp90 make it a potentially viable drug target against several parasitic infectious diseases [15, 16]. Molecular characterization of the PfHsp90 protein from clinical isolates of *P. falciparum* collected from patients in diverse geographical regions has shown that the ATP-binding domain of PfHsp90 is highly conserved among the isolates [17] thus reducing the likelihood of resistance emerging. Hsp90 is an essential protein in eukaryotic systems and not compatible with viability if knocked out. That is, mutations in the ATP binding domain of PfHsp90 make the protein inactive, negatively affecting important biological functions and incur the parasite too much fitness cost [18–20]. Interestingly, it has been shown that PfHsp90 may be associated with a *P. falciparum* chloroquine resistance transporter (PfCRT) protein from a chloroquine-resistant parasite strain. Immunoprecipitation experiments showed that PfHsp90 complex co-immunoprecipitated with PfCRT from *P. falciparum* W2 strain. Moreover, the use of a PfHsp90 inhibitor, PU-H71, resulted in loss of PfCRT protein [21]. It is postulated that targeting PfHsp90 in chloroquine-resistant *P. falciparum* strain could reverse the resistance and render the parasite chloroquine sensitive again. This effect may not be restricted to a single drug class given the broad range of chaperone activity that Hsp90 regulates.

The discovery of the natural compound, geldanamycin opened a new avenue in the development of drugs targeting Hsp90. Geldanamycin has displayed a strong anti-cancer and anti-malarial effect in vitro. However, a strong hepatotoxic signal precludes its clinical use. As a result, different derivatives of geldanamycin have been developed that have an acceptable level of hepatotoxicity [22]. In vitro and in vivo experiments have shown that the geldanamycin-derivatives, 17AAG and 17-PEG-Alkyn-GA, are promising anti-malarial compounds targeting PfHsp90 [23, 24].

In addition to geldanamycin and its derivatives, other small molecules such as purine analogues have been found to effectively inhibit Hsp90 in vitro and in vivo. One such molecule is PU-H71. This molecule has been tested as an anti-cancer drug [25]. Recently, it has been demonstrated that PU-H71 has a good anti-malarial activity in vitro and in mice [21]. Harmine, a beta carboline alkaloid, is able to selectively bind PfHsp90 to a greater extent than human Hsp90 (HsHsp90) [26]. Harmine has been reported as having a specific high-affinity interaction with the ATP-binding domain of PfHsp90 with strong inhibition of *P. falciparum* in cell culture systems. In the *P. berghei* ANKA infection model in mice, harmine showed a significant reduction in parasitaemia. However, it did not significantly prolong the survival of the infected mice [17, 26].

In this study, a library of harmine analogues were generated and their ability to bind PfHsp90, inhibit *P. falciparum* in culture, and kill parasites in the *P. berghei* infection mice model was tested. It was hypothesized that the harmine analogues have a strong anti-malarial effect both in vitro and in vivo in mice. It has been found that some of the harmine analogues effectively bind to PfHsp90, inhibit the growth of *P. falciparum* in vitro, significantly reduce parasitaemia in infected BALB/c mice in a dose-dependent manner, and prolong the survival of infected mice. Interestingly, two daily injections with a combination of 100 mg/kg of 21A (one of the harmine analogues) and 10 mg/kg dihydro-artemisinin (DHA) was able to reduce parasitaemia to an undetectable level in infected mice.

## Methods

### Synthesis of harmine analogues

**Fig. 1:**
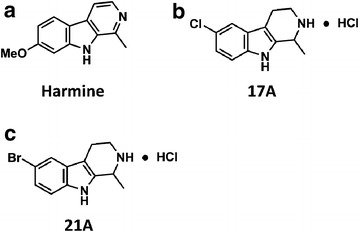
The chemical structure of harmine analogues. Forty-two different analogues of harmine were generated using a microwave-assisted synthetic route. Two compounds were selected based on their binding to the ATP-binding domain of pfHsp90. **a** Harmine, **b** 17A and **c** 21A

Forty-two different analogues of harmine were generated using a microwave-assisted synthetic route, as described previously [27]. Based on the results of the PfHsp90 binding assay, two out of 42 compounds were selected and tested for in vitro and in vivo anti-malarial activity. The chemical structures of harmine 17A and 21A are shown in Fig. 1.

### PfHsp90 competitive binding assay

Binding of the harmine-derived compounds with the ATP-binding domain of PfHsp90 was assessed using a bis-ANS (4, 4′-Dianilino-1, 1′-binaphthyl-5, 5′-disulfonic acid dipotassium salt) binding assay [28, 29]. The expression and purification of the PfHsp90 GHKL domain as well as the bis-ANS assay was performed as previously described [17]. Briefly, the *P. falciparum* Hsp90 GHKL domain DNA was cloned into pET28b plasmid vector and the His-tagged protein was expressed in *Escherischia coli* BL21 (DE3) CondonPlus cells. The recombinant protein was purified using Ni–NTA resin and the His-tag was cleaved using TEV protease. The competitive binding assay was performed by mixing bis-ANS with the purified recombinant protein that was pre-incubated at 37 °C in the presence or absence of the harmine analogues. The fluorescence emission was captured at 490 nm.

### In vitro parasite culture and anti-malarial activity assay

A 72-h in vitro growth inhibition assay was used to test the anti-malarial activity of the harmine analogues. *Plasmodium falciparum* line 3D7 (MRA-102), line W2 (MRA-157), as well as artemisinin-resistant *P. falciparum* strains (MRA-1236 and MRA-1240) were obtained from MR4 (MR4, ATCC^®^ Manassas, Virginia, USA). *Plasmodium falciparum* was cultured in complete RPMI-1640 medium with 1.5% haematocrit and incubated in a CO_2_-enriched atmosphere. The parasite growth was synchronized by treating with 5% sorbitol and cultured for 48 h. In vitro susceptibility of the parasite to the candidate drugs was performed using histidine rich protein (HRP)-II ELISA [30, 31]. First, the drug plates were prepared in ten-fold serial dilution with a total of ten drug concentrations and one drug-free control in 96-well, flat-bottomed Costar^®^ tissue culture plate (Corning, USA) in duplicate. Two-hundred µL of the parasite culture with a parasitaemia of 0.05% and a haematocrit of 1.5% was added into each well with a known drug concentration. The drug-treated parasites were incubated at 37 °C in a candle jar for 72 h. Then, the plates were kept in a −20 °C freezer for at least 24 h before lysis of red blood cells was performed by two freeze–thaw cycles.

HRP-II ELISA was performed following the procedure in the Worldwide Antimalarial Resistance Network (WWARN) manual [32]. Briefly, a 96-well, high-binding ELISA plate (Costar^®^, Corning Inc, USA) was coated with 1.0 µg/mL anti-HRP-II IgM capture antibody (Immunology Consultants Laboratories, OR, USA) in PBS and incubated at 4 °C overnight. Next morning, the unbound antibody was removed by blotting on a paper towel and blocking was performed with 200 µL/well of 2% bovine serum albumin (BSA) at room temperature for 2 h. After removing the blocking buffer, the plate was washed twice with PBS containing 0.05% Tween^®^ 20 (Sigma, MO, USA). One-hundred µL of the sample was then added to each well, incubated at room temperature for 1 h and washed twice as above. Then, 100 µL/well of a secondary anti-HRP-II antibody conjugated with horse radish peroxidase (Immunology Consultants Laboratories, OR, USA) diluted in 2% BSA/1% Tween^®^ 20 in PBS was added and incubated for 1 h at room temperature. The plate was washed twice, and then 100 µL/well of TMB chromogen (Invitrogen, CA, USA) was added and incubated for 2–10 min at room temperature in the dark. The reaction was then stopped by adding 50 µL of 1 M sulfuric acid to each well. Finally, the absorbance at 450 nM was taken using a SpectraMax^®^ 340 ELISA plate reader (Molecular Devices, USA).

### In vivo anti-malarial assay in mice

4- to 5-weeks old female BALB/c mice were purchased from Charles River Laboratories (QC, Canada). The mice were kept in a pathogen-free environment at the health science animal research facility of the University of Calgary, Canada. A review of existing mouse malaria models has been described previously [33]. After acclimatizing for one week, four- to five-weeks old female BALB/c mice were randomly distributed to experimental and control groups. Each group consisted of five mice. *Plasmodium berghei* ANKA (kindly provided by Dr Ian Crandall, University of Toronto, ON, Canada) was thawed at 37 °C water bath and injected aseptically into the donor mice intraperitoneally. Blood was collected by cardiac puncture when the parasitaemia reached 5–10%. Then, 10^6^ parasite-infected red blood cells (RBCs) were injected interaperitoneally into each mouse in the experimental and control groups. After confirmation of parasiatemia, each mouse in the experimental groups was given intraperitoneal injection of the experimental compound(s) dissolved in 10% dimethyl sulfoxide (DMSO). Positive and negative control mice were injected with chloroquine and DMSO, respectively. For the combination experiment, 21A and DHA were prepared separately and mixed just before injection. The efficacy of the drugs was then assessed by determining the percentage of infected RBCs in Giemsa-stained blood collected by tail vein puncture daily. Survival of the drug-treated mice was compared with that of the controls. Mice that showed a net weight loss of more than 20% and with symptoms of severe malaria were euthanized.

### Cytotoxicity assay

Cytotoxicity of the candidate drugs was tested on HepG2 and HeLa cell lines using Cell Counting Kit-8 (Sigma). Immortalized human cell lines such as HepG2 and HeLa cells have been used in previous studies to assess the cytotoxicity of several compounds including anti-malarial candidate drugs in vitro [34–36]. All the cell culture reagents were bought from Life Technologies (Canada). HepG2 cells were cultured in DMEM (low glucose) plus 10% FBS. HeLa cells were grown in DMEM (high glucose) plus 10% FBS and 1 mM Pyruvate.

A modification of the procedure used by Ramirez-Mares et al. [37] was followed. Briefly, exponentially growing cells were trypsinized and suspended in fresh medium at a density of 5 × 10^4^ cells/ml. One-hundred µL of the cell suspension was then dispensed into each well of a 96-well, flat-bottomed Costar^®^ tissue culture plate (Corning, USA) in triplicate and pre-incubated for 24 h in a humidified incubator at 37 °C and 5% CO_2_. Drug plates were prepared by serially diluting the drugs. Eight ten-fold dilutions of each drug were prepared starting at 5 mM. In addition to the candidate drugs, chloroquine, geldanamycin and DMSO were included as controls. Then, 10 µL/well of various concentrations of the drugs was added into the cells. Drug-free cells were also included as a control. The cells were then incubated at 37 °C and 5% CO_2_ for 48 h. Then, 10 µL/well of CCK-8 reagent was added and incubated at 37 °C for an additional 4 h. Finally, the absorbance at 450 nm was measured using a SpectraMax^®^ 340 ELISA plate reader (Molecular Devices, USA). Wells containing only media were also included as a control.

### Statistical analysis

The statistical differences in per cent parasitaemia inhibition between drug-treated and vehicle control mice were analysed using t test. The log-rank (Mantel-Cox) test was done to analyse the statistical difference in the survival of drug-treated and control mice. A *p* value of less than 0.05 was considered statistically significant. All statistical analyses were done using Graphpad Prism 6 software.

## Results

### Harmine analogues bind to PfHsp90

**Fig. 2:**
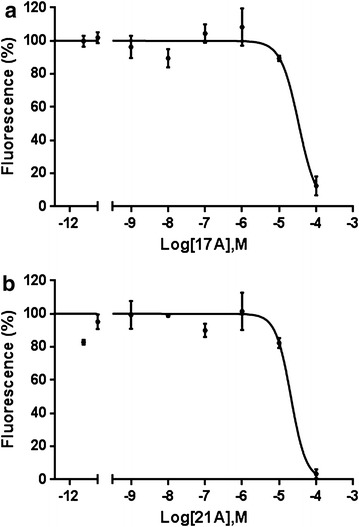
Normalized dose–response curve for the competitive binding assay. Binding of (**a**) 17A and (**b**) 21A to the ATP binding domain of PfHsp90 was assessed using the bis-ANS competitive binding assay. Reduction in the fluorescence of bis-ANS was measured using a spectrophotometer. The average IC_50_ values were calculated from three experiments using Graphpad Prism 6. The mean fluorescence and standard error of the mean (SEM) of duplicate wells is shown. Each graph is a representative of three independent experiments

Of the 42 harmine analogues tested, two (17A and 21A) showed binding to the ATP-binding domain of PfHsp90. The average IC_50_ values for the binding of 17A and 21A with PfHsp90 were 12.2 ± 2.3 and 23.1 ± 8.8 µM, respectively (Fig. 2). Unfortunately, 22 of the harmine analogues (the beta-carboline ‘B’ series of compounds) were not amenable to testing due to auto-fluorescence in the bis-ANS assay and were subsequently excluded from further study.

### Harmine analogues inhibit the growth of *Plasmodium falciparum*

**Fig. 3:**
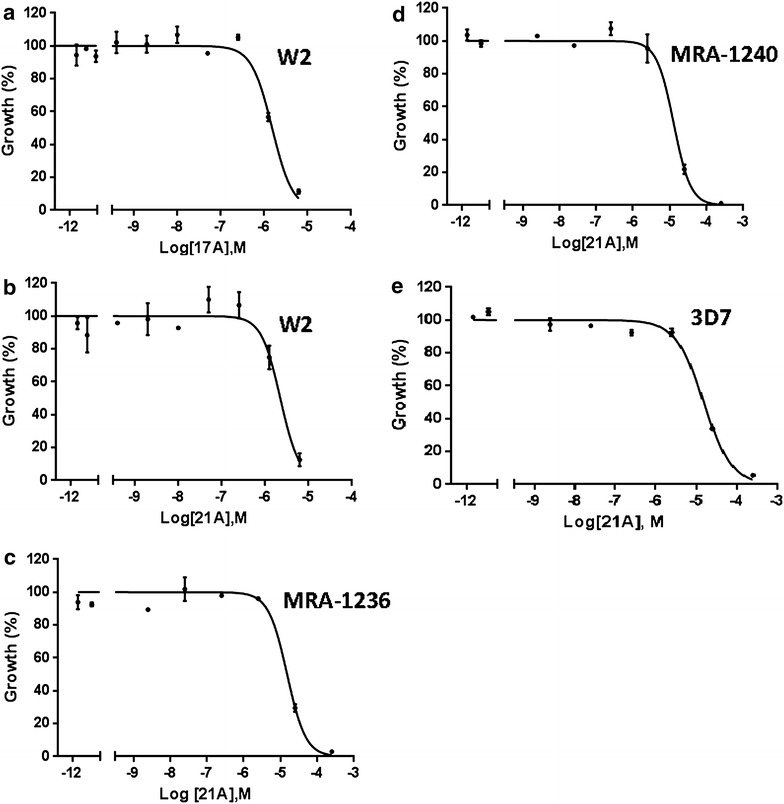
Normalized dose–response curve for the in vitro susceptibility of *Plasmodium falciparum* to 17A and 21A. The in vitro anti-malarial effect of 17A and 21A was measured using HRP-II ELISA of drug treated *P. falciparum* strains. **a** 17A against W2, **b** 21A against W2, **c** 21A against MRA-1236, **d** 21A against MRA-1240, and **e** 21A against 3D7. IC_50_ values were calculated from the dose–response curve using Graphpad Prism 6. The experiment was performed in duplicate wells. The mean OD 450 nm and the standard error of the mean (SEM) of duplicate wells and is shown. Each graph is a representative of three independent experiments

The in vitro anti-malarial efficacy of 17A and 21A was assessed against *P. falciparum* W2 using HRP-II ELISA in a whole cell assay. The average IC_50_ values were 4.2 ± 1.3 and 5.7 ± 1.7 µM for 17A and 21A, respectively. The anti-malarial activity of 21A was also measured against artemisinin-resistant strains. It showed growth inhibition of MRA-1236 and MRA-1240 strains with average IC_50_ values of 9.2 ± 0.4 and 9.6 ± 2.0 µM, respectively. Interestingly, 21A showed comparatively lower activity against chloroquine and artemisinin-sensitive *P. falciparum* 3D7 strain with average IC_50_ value of 13.5 ± 0.8 µM (Fig. 3).

### Harmine analogues significantly reduce parasitaemia in infected mice and prolong their survival

**Fig. 4:**
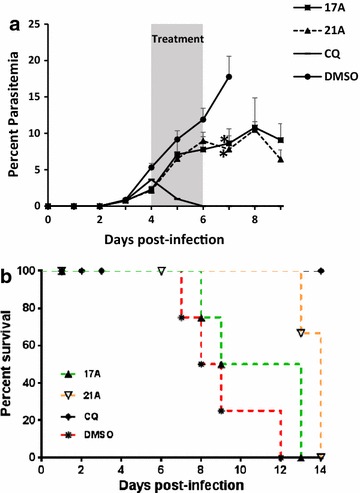
Parasitaemia inhibition and survival of mice treated with a fixed dose of harmine-analogues. Five BALB/c mice per group were infected with *P. berghei* ANKA. The mice were treated with three daily doses of 100 mg/kg of 17A or 21A starting at day-4 post infection. The positive and negative control mice were given three daily doses of 100 mg/kg chloroquine and 10% DMSO, respectively. **a** Per cent parasitaemia. The graph is the mean parasitaemia and standard error of the mean (SEM) of five mice in a group, and **b** Kaplan–Meier survival plot of mice treated with the harmine analogues and controls. *Asterisk* indicates a statistically significant difference between the drug treated mice and the vehicle control group (DMSO)

**Fig. 5:**
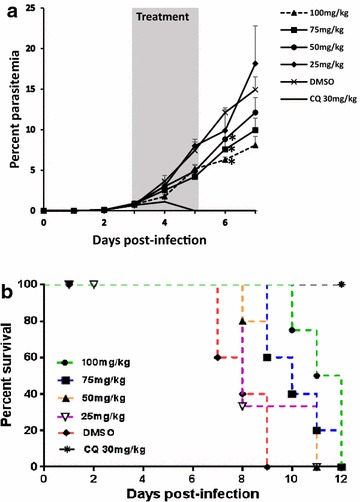
Parasitaemia inhibition and survival of mice treated with different doses of 21A. Five BALB/c mice per group were infected with *P. berghei* ANKA. The mice were treated with three daily doses of 100, 75, 50 or 25 mg/kg of 21A starting at day-3 post infection. The positive and negative control mice were given three daily doses of 30 mg/kg chloroquine and 10% DMSO, respectively. **a** Per cent parasitaemia. The graph is the mean parasitaemia and standard error of the mean (SEM) of five mice in a group, and **b** Kaplan–Meier survival plot of mice treated with different doses of 21A and controls. *Asterisk* indicates a statistically significant difference between the drug treated mice and the vehicle control group (DMSO)

The in vivo efficacy of the harmine analogue compounds was tested using *P. berghei* ANKA infection in BALB/c mice. For the fixed-dose experiment, the drug treatment was started at day-4 post-infection with a starting average parasitaemia of about 3.5%. Three daily injections of infected mice with 100 mg/kg of each of 17A and 21A showed statistically significant reduction in parasitaemia compared to the vehicle control (p < 0.05). At day-7 post-infection (i.e., one day after the last dose of the drugs), 17A and 21A resulted in reduction of parasitaemia by 51.5 and 56.1%, respectively (Fig. 4a). The positive control mice that received a daily injection of 30 mg/kg chloroquine for three consecutive days cleared the infection to microscopically undetectable levels and remained so until the end of the experiment. Figure 4b shows the Kaplan–Meier survival curve of the mice treated with the candidate drugs and controls. Mice treated with 17A and 21A showed a median survival time of 11 and 14 days, respectively, while the vehicle control mice showed a median survival time of only 8.5 days. Log-rank (Mantel-Cox) test indicated that the survival of mice treated with 21A was significantly higher than vehicle control mice (p < 0.05). Of the two hamine-analogues tested for in vivo anti-malarial activity at a fixed dose, 21A was selected for a dose-ranging experiment. Four different doses of 21A were used for the dose-ranging experiment; 100, 75, 50 and 25 mg/kg. Chloroquine and DMSO were used as a positive and vehicle controls, respectively. Five female BALB/c mice were used in each group. The degree of parasitaemia and survival rate in infected mice showed that 21A has anti-malarial activity in a dose-dependent manner. One day after administration of the third dose, mice treated with 100, 75 and 50 mg/kg demonstrated a significantly lower parasitaemia than the mice in the vehicle control group (p < 0.05) (Fig. 5a). The per cent parasitaemia reduction in these groups was 48.1, 37.5 and 27.2%, respectively. Like in the fixed-dose experiment, mice that were treated with chloroquine cleared the infection to microscopically undetectable levels. Comparison of drug-treated mice with the vehicle control ones indicated that at doses of 50 mg/kg and above, the survival of drug-treated mice increased significantly than the controls (p < 0.05) (Fig. 5b).

## 21A has additive effect with dihydro-artemisinin

**Fig. 6:**
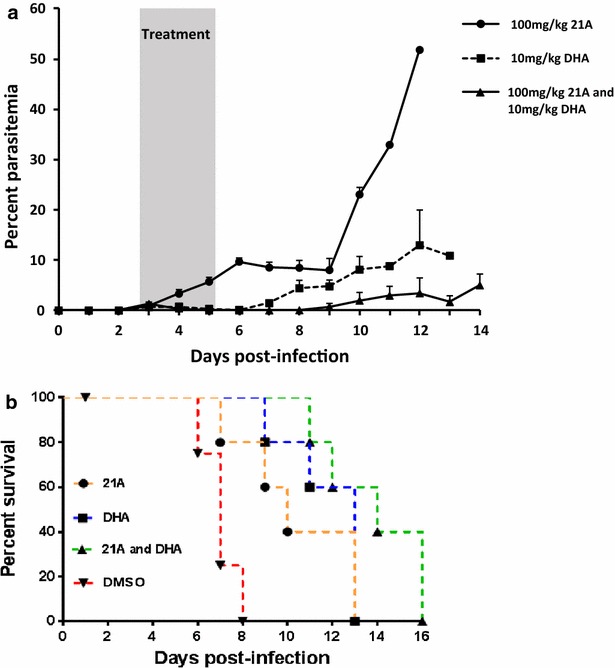
Parasitaemia inhibition and survival of mice treated with a combination of 21A and dihydro-artemisinin. Five BALB/c mice per group were infected with *P. berghei* ANKA. The mice were treated with three daily doses of 100 mg/kg 21A, 10 mg/kg DHA, a combination of 100 mg/kg 21A and 10 mg/kg DHA starting at day-3 post infection. The negative control mice were given three doses of 10% DMSO. **a** Per cent parasitaemia. The graph is the mean parasitaemia and standard error of the mean (SEM) of five mice in a group, and **b** Kaplan–Meier survival plot of mice treated with 21A, DHA, and a combination of 21A and DHA

In order to investigate the potential of 21A to be used as a partner drug with artemisinin in a combination therapy model, the anti-malarial efficacy of a combination of 21A and DHA was tested and compared—with the efficacy of the individual drugs alone. The data show that treatment with a combination of 100 mg/kg 21A and 10 mg/kg DHA results in a more potent anti-malarial activity than treatment with DHA alone. Figure 6a shows the per cent parasitaemia inhibition of treatment with a combination of 21A and DHA or with individual drugs alone. One day after administration of two doses of the combined drugs, all five mice (100%) cleared the infection with undetectable parasitaemia by microscopy. In contrast, only 40% (two out of five) of the mice treated with DHA alone cleared the infection at this time point. No mouse treated with 21A alone achieved undetectable parasitaemia. One day after the last dose of treatment, 100 and 60% of the mice that received the combination and DHA alone had undetectable parasitaemia, respectively (Fig. 6a). However, the mice that received the combination developed microscopically detectable parasitaemia at later time points. Likewise, the mice treated with a combination of 21A and DHA survived longer than those that were treated with either 21A or DHA alone. Two mice survived until the end of the experiment (day-16) and were sacrificed (Fig. 6b).

## 17A and 21A are not toxic to HepG2 and HeLa cells

**Fig. 7:**
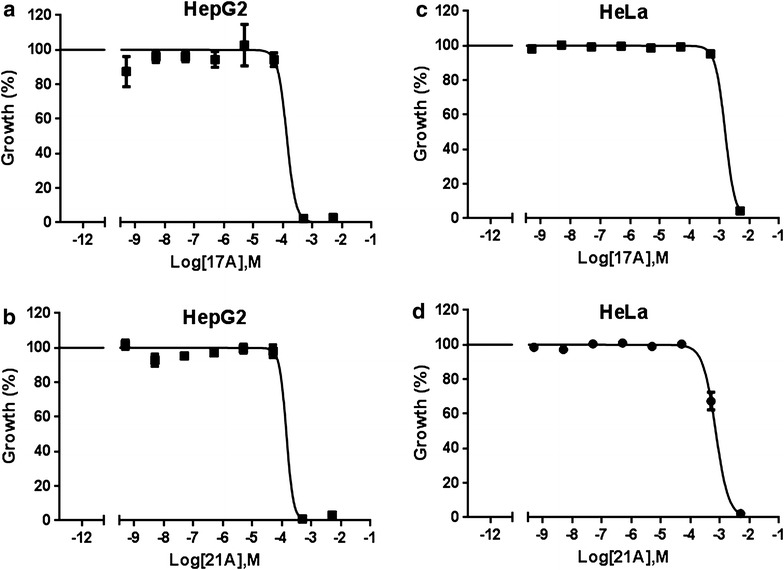
Normalized dose–response curve for in vitro cytotoxicity of 17A and 21A. Human cell lines were cultured in the presence of 17A and 21A. Cytotoxicity of the compounds was evaluated using the CCK-8 kit. Per cent cell death was measured by measuring absorbance at OD 450 nm. **a** HepG2 cells treated with 17A, **b** HepG2 cells treated with 21A, **c** HeLa cells treated with 17A, and **d** HeLa cells treated with 21A. The mean OD 450 nm and the standard error of the mean (SEM) of triplicate wells and is shown. Each graph is representative of three independent experiments

To investigate the possibility of toxicity in human host, the cytotoxicity of the compounds was tested in vitro in HepG2 and HeLa cells. The result showed that 17A and 21A are not toxic to either cell line. In HepG2 cells, the IC_50_ values of 17A and 21A were 0.16 ± 0.01 and 0.140 ± 0.001 mM, respectively. On the other hand, in HeLa cells, 17A and 21A had IC_50_ values of 1.1 ± 0.3, 0.48 ± 0.14 mM, respectively (Fig. 7). Since it has proven hepatotoxicity, geldanamycin was also included as a control. As expected, it showed high degree of toxicity in HepG2 cells with IC_50_ in micromolar range (0.34 ± 0.13 µM).

## Discussion

Heat shock proteins are crucial molecular chaperones that are involved in various central metabolic activities of both prokaryotic and eukaryotic cells. Because Hsps are necessary for normal metabolic activities of a cell and to protect cells from stress conditions, they are conserved in all life forms. Although they are constitutively expressed, the level of expression of Hsps increases when challenged by stressors such as heat or pH changes. Hsp90’s crucial role as an essential chaperone, association with resistance, and high degree of conservation make it an attractive adjunctive drug target [18, 19, 38, 39].

As a parasite with a life cycle involving poikilothermic insects and homoeothermic humans, *P. falciparum* has to adapt to the drastic change in temperature during transmission from the insect vector to human host. *Plasmodium falciparum* Hsp90 protein is the most abundant cytosolic protein and plays an indispensable role in resisting the effect of temperature change that occurs during transmission from insect vectors to humans.

The ATPase activity of Hsp90 is a necessary precursor to its function and interaction with client proteins. Therefore, targeting the Hsp90 ATP-binding using a small molecule is expected to inhibit its chaperoning activity and render the client proteins to degradation via the cytosolic proteasome, which consequently inhibit major metabolic activities of the cell [40]. Several studies have tested the use of small molecules that bind to and competitively inhibit the ATPase activity of Hsp90 as candidate drugs for different infectious diseases and cancer [23, 41, 42]. Although the Hsp90 in humans and the malaria parasite have a high degree of homology, there are subtle and potentially significant structural differences that can be exploited when designing selective small-molecule inhibitors [16]. Moreover, cells from different organisms show variable degree of dependency on chaperone-supported metabolic activities. As a result, it is hypothesized that it is possible to inhibit the activity of Hsp of a pathogen without significant deleterious effects on the Hsps of the host [5, 17]. It has been shown previously that natural compounds such as harmine and the ATP mimetic PU-H71 exert anti-*Plasmodium* activity by targeting PfHsp90 [21, 26]. In this study, 42 different derivatives of harmine were synthesized and the in vitro and in vivo anti-malarial activities of two of the harmine analogues was tested as single agents and in combination with artemisinin. Unfortunately, 22 harmine analogues displayed auto-fluorescence in bis-ANS assay and were consequently excluded from the study.

Two out of the remaining 20 compounds (17A and 21A) effectively bind to the ATP-binding domain of PfHsp90 with IC_50_ value in the low micromolar range. The IC_50_ values of 17A and 21A was comparable to that of radicicol, a compound that has been shown to tightly bind Hsp90 [43].

In vitro susceptibility of 21A showed that the compound has anti-malarial activity against both chloroquine- and artemisinin-resistant *P. falciparum* strains with micromolar IC_50_ value. Generally, the chloroquine- or artemisinin-resistant strains seem more susceptible to 21A than the chloroquine-sensitive 3D7 strain. Similar effect was seen in the previous study with harmine. That is, chloroquine-resistant W2 strain was almost twice more susceptible to harmine than chloroquine-sensitive 3D7 strain [17]. It remains to be seen if inhibition of PfHsp90 in chloroquine-resistant strains affects other metabolic activities of the parasite in addition to reversing the resistance trait. The effect of inhibition of PfHsp90 activity on proteins associated with chloroquine- or artemisinin-resistance needs further study.

At a fixed dose of 100 mg/kg administered daily for three consecutive days, both 17A and 21A showed a significant parasitaemia reduction. Unlike 17A, treatment with 21A significantly prolonged the survival time of treated mice. This property was not seen with the parent molecule harmine [26]. Therefore, 21A was selected for the dose-ranging and combination experiments. 21A showed a dose-dependent activity with the highest parasitaemia reduction at a dose of 100 mg/kg followed by 75 and 50 mg/kg. One day after the last dose of treatment, the group treated with 25 mg/kg did not show significant difference from the vehicle control. Interestingly, 21A showed an additive effect with DHA in vivo in mice. Treatment with a combination of 100 mg/kg 21A and 10 mg/kg DHA resulted in a dramatic reduction of parasitaemia to undetectable levels by microscopy on Giemsa-stained peripheral blood in just two doses. This stands in stark contrast with the result from mice that were treated with DHA alone where only two out of five mice cleared the infection. As such, 21A has promising potential as a partner drug with artemisinin either in combination or co-formulation. This is in line with previous studies that showed the possibility of using Hsp90 inhibitors as adjunctive drugs with the current anti-malarial drugs [39]. Of note, it is not possible to determine if the combined effect in mice was due to synergy or an alternative effect. Because the parasitaemia in the combination treatment groups was reduced to undetectable levels by microscopy, it was not possible to calculate whether 21A has synergistic effect with DHA in vivo. FACS analysis of DNA-stained RBCs could have been an alternative approach to determine the parasite load in such conditions. However, FACS analysis was not performed due to lack of the required laboratory facility. On the other hand, pharmacokinetic analysis of 21A and DHA in mice was not performed in this study. Pharmacokinetic and drug–drug interaction study between 21A and DHA is warranted to better understand the synergy.

In this study, differences were seen in the parasitaemia of mice that were treated with the same amount of 21A in different experiments. This could be probably due to differences in experimental conditions. One such factor is the difference in the initial parasitaemia before drug administration.

In order to be considered as an effective candidate drug, a compound should not be toxic to the human host. The first step in evaluating toxicity is to investigate the cytotoxicity in cell lines in vitro. In this regard, the cytotoxicity of 17A and 21A was tested in HepG2 and HeLa cells in vitro. Comparing the IC_50_ value for the cytotoxicity with that of in vitro anti-malarial activity, in HepG2 cells, 17A and 21A had a favourable cytotoxicity selectivity index (SI) of 38 and 25, respectively. In HeLa cells, 17A and 21A had an SI of 259 and 84, respectively. Future studies require the pursuit of a second generation library based on the current data which probably will yield compounds with increased potency against malaria in all stages of the life cycle.

## Conclusion

The emergence of *P. falciparum* strains that are resistant to artemisinin or to the partner drugs in ACT poses a serious challenge to the current malaria elimination agenda. This calls for the development of new ‘smart’ drugs that could be used as partner drugs with artemisinin. It has been previously shown that *P. falciparum* Hsp90 is highly conserved and performs a fundamental chaperone role in the cell. Harmine analogues that inhibit PfHsp90 have been developed and their anti-malarial activity tested in vitro and in vivo in mice. Two harmine analogues, 17A and 21A, showed in vitro anti-malarial activity with micromolar range IC_50_ values. The analogues also showed in vivo anti-malarial activity with significant parasitaemia reduction in infected BALB/c mice and increased survival. More importantly, 21A showed additive effects when used in combination with DHA, indicating that 21A could be potentially used as a partner drug candidate. Future study on the pharmacokinetics of 21A and also drug–drug interaction with DHA would be of great importance.

## Abbreviations

PfHsp90: *Plasmodium falciparum* heat-shock protein 90
ACT: artemisinin-based combination therapy
DHA: dihydro-artemisinin
PfCRT: *Plasmodium falciparum* chloroquine resistance transporter
BSA: bovine serum albumin
HRP-II: histidine-rich-protein-II
PBS: phosphate buffered saline
DMSO: dimethyl sulfoxide
ELISA: enzyme-linked immunosorbent assay
